# War-Related Stress among Israeli College Students Following 7 October 2023 Terror Attack in Israel

**DOI:** 10.3390/ejihpe14080145

**Published:** 2024-07-30

**Authors:** Keren Dopelt, Nourit Houminer-Klepar

**Affiliations:** Department of Public Health, Ashkelon Academic College, Ashkelon 78211, Israel

**Keywords:** 7 October terror attack, stress, trauma, Hamas–Israel war, Iron Swords war, sleep quality, students, Israel

## Abstract

Background: Warfare represents a significant source of stress in contemporary times, with enduring implications beyond the immediate casualties, fostering a pervasive atmosphere of danger and anxiety within affected populations. The Israel–Hamas war, marked by ongoing armed incursions and missile attacks, stands as a recent example of such turmoil, inflicting widespread trauma and disruption. Methods: This study, conducted among students at the Ashkelon Academic College in southern Israel, aimed to investigate the stress levels and associated factors amidst the ongoing conflict. Utilizing a cross-sectional survey design, data were collected from 625 participants between January and February 2024, approximately four months after the commencement of the initial attack. The survey encompassed demographic information, perceived stress levels, sleep quality, and social media usage. Results: The findings revealed moderate to high stress levels among participants, with significant differences observed based on gender, parental status, and residency in conflict zones. Moreover, poorer sleep quality was reported among students residing in conflict-affected areas. Regression analysis identified several predictors of elevated stress, including gender, parental status, sleep quality, residency in conflict zones, and social media usage. Conclusions: These findings underscore the profound impact of ongoing conflict on college students’ mental well-being, highlighting the need for tailored interventions and support services within higher education institutions. The limitations include the study’s focus on a specific college population and the timing of the data collection relative to the onset of the war. Nonetheless, this research contributes valuable insights concerning the stress dynamics within the unique context faced by Israeli students amidst ongoing warfare.

## 1. Introduction

### 1.1. Background

In contemporary times, warfare stands out as a significant source of stress [1]. Since the establishment of the State of Israel, it has been involved in a series of wars, conflicts, and persistent invasion attempts by both Arab nations’ militaries and terrorist organizations [2]. The repercussions of warfare and conflict frequently surpass the immediate casualties, fostering a pervasive atmosphere of danger, anxiety, and related psychological distress within the broader population [3].

On 7 October 2023, Hamas and several other Palestinian militant groups launched coordinated armed incursions from the Gaza Strip into the Gaza envelope of southern Israel. Since the attacks commenced, the Israel–Hamas conflict has persisted. From 7 October onwards, thousands of missiles have been fired at Israel from different directions: Gaza, Iran, Lebanon, Yemen, Syria, and Iraq. More than 1600 Israelis have been killed, hundreds of civilians were kidnapped, tens of thousands were injured, hundreds of thousands of individuals were forced to evacuate their homes, and an entire nation has been seeking refuge in shelters for an extended duration. It stands as the most lethal assault on Jews since the Holocaust, inflicting individual and collective trauma and stress upon the citizens of the State of Israel and Jewish communities worldwide.

### 1.2. Factors Associated with Stress during Warfare

Stress represents a psychological disruption that may arise from the trauma of war [4]. In the realm of academia, college students face unique stressors [5,6,7,8,9,10]. During wartime, this situation may be exacerbated [11]. In the present context of Israel, young individuals are burdened by numerous fears and stressors. Since the onset of war and the terrorist attacks on 7 October, these have emerged as predominant stress triggers. Stress can manifest in various forms among students, including diminished mental acuity, persistent fatigue, and poor concentration in classrooms. Despite often experiencing anxiety, students may not always seek assistance [12]. 

Zhihaylo et al. [12] identified the following factors triggering stress amid wartime conditions: heightened anxiety, sleep disturbances, fear or panic during raids and while in bomb shelters, concern for personal safety, mood swings, decline in memory function, decreased attention and concentration levels, learning difficulties, and reduced inclination to engage in social interactions. The researchers found heightened levels of anxiety (18%), sleep disturbances (63%), and mood swings (63%) among students in Ukraine. They referenced the Ukraine Ministry of Health’s assertion that the psychological repercussions of the Russian–Ukraine war will continue to impact Ukrainians’ mental well-being for a period spanning at least 7–10 years following the end of the war. 

Direct exposure to warfare significantly heightens the associated anxiety and depression disorders, along with an increased perception of stress. Research investigating the symptoms of post-traumatic stress disorder (PTSD) suggests that the ramifications of trauma can persist over an extended period, spanning years [13,14]. Furthermore, exposure to multiple traumatic events amplifies stress levels [15]. Direct exposure to stressors related to war significantly increases the manifestation of symptoms associated with anxiety and depression disorders, as well as perceived stress [16]. According to Kurapov et al. [17], Ukrainian students are experiencing a notable decrease in their psychological well-being. The study revealed that 97.8% of respondents reported experiencing exacerbated psycho-emotional symptoms, including increased levels of depression, loneliness, nervousness, and anger, attributed to the ongoing Russian–Ukraine war. Pavlenko et al. [18] examined women’s mental health in the education sector, including both students and faculty members during the Russian–Ukraine war. Their study revealed a heightened prevalence of fear and stress among the respondents. Notably, individuals who have endured the strains of war may see a gradual deterioration in their mental health over time [19]. Arheiam et al. [20] conducted a comparative analysis of the stress levels among dental students in Libya, differentiating between those residing in conflict-free regions and those in war-affected zones. Their findings indicated that individuals living in conflict zones experienced significantly higher levels of associated stress. In addition, a higher prevalence of war-related stress is observed among females compared to males [16,20,21,22]. Additionally, the impact of conflict on sleep quality is notable, with studies suggesting that experiences of war contribute to sleep disturbances, particularly in conjunction with mild traumatic brain injury [23].

Stress is also intertwined with the pervasive influence of social media, which shapes individuals’ perceptions, emotions, and behaviors [24]. The influence of social media on individuals’ mental health, particularly in wartime, cannot be overlooked [25]. In the context of the ongoing war, social media platforms serve as critical channels for spreading information, shaping public communication, and fostering connections among individuals [26]. However, the constant exposure to distressing news updates, graphic images, and online debates can exacerbate feelings of anxiety, uncertainty, and fear among users, contributing to heightened stress levels. Moreover, the interplay between social media usage and stress during wartime is complex, with individuals often turning to online platforms for support, solidarity, and information-seeking, yet inadvertently exposing themselves to triggering content that amplifies their distress [27]. 

### 1.3. Research Questions

The majority of prior research has focused on examining the psychological and mental well-being of communities affected by conflict or veterans during the post-war periods (e.g., [22,28,29,30]). Research on the psychological impacts of war on civilians during active conflict periods remains notably sparse. Even scarcer are studies exploring the stress experienced by young adults, including college students, who have spent most of their lives amidst ongoing armed conflict. Disruptions to life trajectories, experiences of loss, and distress from shelling have the potential to trigger stress disorders among Israeli college students. Therefore, the primary objective of this study was to investigate the following research questions:

1. What is the prevalence and severity of war-related stress among Israeli college students four months after the commencement of the war?
2. Do differences in stress levels exist between students residing in conflict-affected areas (close to the border) and those in non-conflict areas?
3. Can socio-demographic factors, including age, gender, religion, sleep quality, and time spent on social media, serve as predictors of stress levels?

The current study was conducted among students at the Ashkelon Academic College in southern Israel, located 14 km (8 miles) from the Gaza Strip. In 2024, the college accommodated approximately 4200 students amid a notable period of regional conflict. Some of these students had to cope with the loss of loved ones. Some joined the military and engaged in combat, while others were compelled to evacuate their homes. The city of Ashkelon has endured frequent rocket fire since 2000, contributing to the heightened stress and anxiety among its residents [31]. Despite this research being conducted four months after the initial attack on 7 October, Israeli citizens continue to face the ongoing threat of existential multi-arena warfare and trauma. The findings of this study may aid college leadership in devising interventions to support students during periods of national crisis.

## 2. Materials and Methods

### 2.1. Procedure

This study adopted a cross-sectional survey design and received ethical approval from the Ashkelon Academic College Ethics Committee (approval #47-2024). Utilizing Qualtrics (Qualtrics, Provo, UT, USA), the survey questionnaire was distributed to all the students by email via the college’s computing infrastructure. The study was conducted between January and February 2024. The software data indicated an average response time of approximately 5 min for the questionnaire. A total of 868 entries were recorded, with 783 students commencing the questionnaire. Among them, 625 students completed the questionnaire. Consequently, the response rate accounted for 72% of the total survey entries and 15% of the research population. The introduction page clarified the study’s objectives and assured participants of anonymity. By completing the questionnaire, students demonstrated their voluntary agreement and informed consent to participate. Participants were free to discontinue their responses at any point, and there was no obligation to answer specific questions.

### 2.2. Participants

A total of 625 students participated in this study, with 72% being women, 51% being in relationships, and 27% having children. Most participants were Jewish (80%), and 42% resided in the conflict zone. More than half of the students studied in the Faculty of Social Sciences (57%), 26% in Health Sciences, and 17% in Computer Science and Management. The mean age of the participants was 27.90 ± 9.01 years (range 18–60). No significant differences were found between students who live in conflict zones and students who do not regarding their demographic characteristics. 

Over half of the participants reported good sleep (54%), with the remaining reporting poor sleep (46%). The average number of sleeping hours was 6.32 ± 1.42 years (range 2–10). The demographic composition of the survey population resembles the college population with regard to gender, age, and faculty composition. The sample’s characteristics are summarized in Table 1.

### 2.3. Tools

We utilized an anonymous, online, closed, self-administered questionnaire. To assess the clarity of the questions, we administered the questionnaire to ten students unaffiliated with Ashkelon Academic College. Furthermore, the questionnaire underwent content validation by three experts: a public health researcher, a sociologist, and a psychologist.

The questionnaire consisted of the following three parts:

1. The demographic and general information collected encompasses gender, age, marital status, religion, department, residence in the conflict zone (yes/no), and estimated average daily time spent on social media.
2. War-related stress was assessed via the Perceived Stress Scale (PPS) [32]. The questionnaire assesses an individual’s level of perceived stress. It consists of 10 items. Six items relate to general feelings of stress and strain experienced by the individual. An additional four items examine the extent to which the individual feels capable of coping with these feelings. Responses range from 1–5 (1—“Never”, 5—“Often”). The overall score is based on the average of all the items, with a higher score indicating a higher stress level. The questionnaire has been translated into Hebrew and validated, and it has demonstrated reliability in multiple studies. The original questionnaire instructed participants to reflect on the month before the survey. We modified the instructions to “since the beginning of the war”. The reliability in the current study was Cronbach’s α = 0.87, indicating high internal consistency.
3. The sleep quality assessment comprised two statements: How many hours did you sleep last night (sleeping hours, not hours spent in bed)? Rate the quality of your sleep on a scale of 1–4. The statements were taken from the Pittsburgh Sleep Quality Index (PSQI), which assesses sleep habits during the previous month [33]. This study utilized the self-reported sleep quality and duration components of this index. A previously translated and validated Hebrew version of the PSQI yielded an internal consistency reliability of Cronbach alpha = 0.72 [34].

### 2.4. Data Analysis

The data were analyzed using SPSS 29.01 (IBM, Armonk, NY, USA). The relationships between age and time spent on social media and stress were tested using the Pearson correlations. The relationship between sleep quality and stress was tested using the Spearman correlation. Gender, marital status, religion, and residency in the conflict zone were compared using independent samples *t*-tests, while differences between faculties were assessed using one-way analyses of variance (ANOVA). A multiple linear regression model was used to predict the level of stress. Differences between students who live in the conflict zone and those who do not in terms of sleep quality were tested using the χ^2^ test. The model included variables that were linked with stress in the univariate analyses. The model incorporated gender, parental status, age, sleep quality, residency in conflict zones, and time spent on social media. Significance in terms of the reported *p*-values was determined through two-sided tests, in which values below 0.05 were considered significant.

## 3. Results

### 3.1. War Stress

The distribution of responses to the questionnaire assessing stress levels amidst the war is presented in Table 2. The responses were grouped as follows: answers 1 and 2 were merged into the category “seldom”, answer 3 was categorized as “sometimes”, and answers 4 and 5 were combined into the category “often”.

The stress level was assessed by computing the mean for each student, resulting in a value of 2.97 ± 0.74. Following the PSS questionnaire guidelines, 120 participants (19%) fell into the low-stress category, 397 participants (63%) were classified as experiencing moderate stress, and the remaining respondents (n = 108, 18%) were categorized as experiencing high stress.

### 3.2. Living in the Conflict Zone and Stress

We examined the differences between students who live in the conflict zone and students who do not regarding their stress levels using the independent samples *t*-test. We found that students residing in the conflict zone report heightened levels of stress compared to those in non-conflict zones (mean 3.08 ± 0.77 vs. 2.89 ± 0.72 respectively, t_(623)_ = 2.98, *p* < 0.01).

### 3.3. Relationships between Socio-Demographic Factors, Sleep Quality, Social Media Usage, and Stress

Table 3 presents the variations in stress levels across different groups, as well as the relationships between age, sleep quality, and time spent on social media and stress. The differences were examined using independent samples *t*-tests or one-way ANOVA according to the scale of the independent variable. The relationships between variables were tested using the Pearson and Spearman correlations depending on the variables’ scale.

This study revealed significant differences in stress levels across student demographics. Female students reported higher levels of stress compared to males. Additionally, students with children reported greater stress levels in comparison to students without children. 

Moreover, statistically significant, moderate negative correlations were observed between age and sleep quality in relation to stress (r_p_ = −0.12, *p* < 0.05; r_s_ = −0.22, *p* < 0.001, respectively), indicating that younger students and those with poorer sleep quality reported higher stress levels. Daily social media usage exhibited significant, moderate, and positive correlations with stress (r_p_ = 0.23, *p* < 0.001). This implies that students who devote more time to social media tend to experience higher stress levels.

In addition, we examined the differences between students who live in the conflict zone and students who do not in terms of sleep quality using the χ^2^ test. We found that students residing in the conflict zone report poorer sleep quality compared to those in non-conflict zones (51% vs. 42%, χ^2^ = 5.40, *p* < 0.05).

### 3.4. Predictors of Stress—A Regression Model 

A linear regression model was employed to identify significant predictors of stress in college students. The model included gender, parental status (having children or not), age, sleep quality, residency in conflict zones, and time spent on social media. The analysis revealed that several factors were significantly associated with higher stress levels (*p* < 0.001), such as being female, having children, experiencing poorer sleep quality, residing in a conflict zone, and spending more time on social media. These variables were identified as predictors of elevated stress. The model accounted for 19% of the explained variance (*p* < 0.001). The detailed results are presented in Table 4.

## 4. Discussion

This study investigated stress among college students in Israel during wartime. Understanding stress in this context is crucial, given the distinct stressors students encounter while studying and residing in proximity to conflict zones. Significant disasters, like the 7 October terror attack, evoke distress among individuals directly impacted and have a ripple effect on the broader community. The comprehension of such situations often leads to heightened levels of anxiety and apprehension, resulting in increased stress levels for both individuals and society at large [35]. 

Our analysis revealed that 18% of participants fell into the high-stress group, 63% were categorized as moderate stress, and 19% were classified as low stress. These findings are concerning when compared to similar studies since students worldwide typically report lower stress levels. For example, Beiter et al. [9] reported that among 374 undergraduates surveyed in Ohio, USA, 11% reported symptoms of severe stress, 27% reported moderate stress, and 62% reported low stress levels. Similarly, in a study of 304 students in Australia, 82% reported mild stress levels, while 18% reported moderate/severe/extremely severe stress levels [36]. In a national survey among 6032 university students in China, the number of students with various stress complaints accounted for 12%. Amongst the different stress levels, mild stress levels accounted for 48% of participants, whilst very severe complaints of stress accounted for 6% of students [37]. Among 1074 undergraduate students in Spain, 66% exhibited no symptoms of stress, while 12% reported mild stress levels, and 24% reported moderate/severe/extremely severe stress levels. Notably, women reported experiencing any level of stress significantly more than men (41% vs. 19%, respectively) [38]. The impact of war as a stressful life event [39,40] may elucidate the heightened stress levels observed among students compared to their counterparts in other Western countries. This study identified significant differences in stress levels based on student demographics, with female students reporting higher stress levels than males. These findings are consistent with prior research [16,20,21,22,41]. Rugema et al. [42] emphasized the prevalence of depression, stress, anxiety, and suicide attempts in Rwanda, particularly among women, whose rates are twice as high as those in men. They attributed this discrepancy to women’s heightened exposure to physical and sexual abuse, which exacerbates their mental health difficulties.

Additionally, students with children reported greater stress compared to their childless student counterparts. The literature highlights the importance of familial social support [43,44,45]. Notably, Fel et al. [44] revealed a correlation between parenthood and increased susceptibility to severe stress, causing significant psychological strain, particularly evident among participants in conflict situations. Similarly, Van Der Feltz-Cornelis et al. [46] established a positive association between parenthood and elevated stress levels. The context of war further exacerbates mental distress, instilling fear regarding the safety and future prospects of one’s children, alongside concerns about providing them with secure living conditions, particularly in conflicts such as those involving missile attacks on civilian populations in regions like Israel. It is reasonable to infer that parents experience heightened anxiety regarding the well-being of their offspring.

Students who live in the conflict zone reported higher stress levels compared to students who do not live in the conflict zone. These findings are consistent with other studies conducted on Libyan students [20], students in Ukraine [17], Ukrainian veterans and active-duty military personnel [47], and Syrian refugees in Turkey [48].

In addition, we found a positive relationship between stress levels and sleep quality. Moreover, students residing within conflict zones reported notably lower sleep quality than their counterparts living in non-conflict areas. The impact of conflict on sleep patterns has been extensively documented [23,49]. For example, during the 1991 Gulf War, Askenasy and Lewin [50] conducted a study involving 1045 individuals surveyed both during and after the conflict, followed by a second interview with the same participants (excluding chronic insomniacs) 30 days post-war. They discovered a significant disruption to sleep patterns. Specifically, 51% of participants reported disturbed sleep during the conflict, with a marked increase in insomnia cases from 13% before the war to 38% during it. Various symptoms, such as stress (67.5%), depressed mood (50.9%), concentration difficulties (39.7%), and increased fatigue (25%), were prevalent during the war. Even four weeks after the war, 19% of previously normal subjects still suffered from insomnia, and 5% developed it afterward. Notably, stress, depressed mood, and impaired concentration were significantly associated with insomnia. They concluded that modern missile warfare may lead to enduring insomnia in approximately one-third of the affected population, with a small percentage developing it post-war. Prolonged stress and depressed mood were identified as risk factors for persistent insomnia. In line with these findings, the present study identified poor sleep quality among 46% of participants. Moreover, Houminer Klepar et al. [51] found that students experiencing high levels of war-related stress were more susceptible to emotional eating disorders, underscoring the multifaceted impact of conflict-related stressors on individuals’ well-being.

The findings regarding the positive correlation between social media usage and stress are also notable, given that participants (including students in general) spend many hours each day on social networks, exposed to news, harsh videos, and difficult-to-process information. Since 7 October, horrifying videos from the massacre on that day have been circulating on social media, along with videos of anti-Semitic protests worldwide, contributing to increased feelings of existential threat, stress, and anxiety. Our findings are concerning in light of the fact that individuals who experience acts of political violence and terror face a heightened risk of experiencing adverse long-term consequences [52]. Currently, every Israeli is contending with feelings of uncertainty and fear regarding their loved ones and the nation, leading to increased levels of stress. Psychologically, stress responses to crisis situations can occasionally serve as a protective mechanism for the body. Nevertheless, it is essential to take prompt measures to mitigate stress and forestall the emergence of more severe consequences [53].

The findings of this study have significant implications for the adaptation of coping strategies within higher education institutions, particularly emphasizing the need to cultivate effective coping mechanisms for students during periods of conflict. Higher education institutions can offer psychological support services during crises and lessen the need for academic achievements during crises to enhance students’ well-being. Academic performance is a significant source of stress and anxiety; measures such as workload reduction, abbreviated study days, and scheduled breaks can assist students in managing their tasks amidst wartime conditions. Providing support, particularly to students actively involved in combat, is of paramount importance. Ecological models offer comprehensive approaches to trauma recovery, suggesting that the success of interventions hinges on their ability to enhance the bond between the individual and the community [41]. Overemphasizing medical factors while disregarding other social dimensions (cultural, political, or economic influences) contributing to stress can be misleading. Therefore, a comprehensive approach, considering both health-related and social factors, will facilitate the development of interventions and bolster support systems for individuals within their familial, collegiate, and broader social networks.

According to Lunov and Rozhkova [54], during times of war, students’ mental resilience undergoes significant strain, necessitating tailored support strategies within higher education settings. Establishing safe platforms for open discussion of their concerns and fears is crucial for fostering psychological well-being. Colleges must prioritize inclusivity and kindness, encouraging a balanced daily routine that integrates academic responsibilities with self-care. Workshops promoting mindfulness, yoga, and stress reduction can strengthen coping mechanisms while engaging in volunteer activities and build community resilience. Addressing campus safety and emergency preparedness reduces anxiety, alongside proactive faculty support and mental health referrals. Cultivating a growth mindset and providing accessible counseling services normalize help-seeking behaviors, reinforcing a supportive learning environment where students can navigate adversity and thrive.

### Study Limitations

The current study was confined to students at Ashkelon Academic College, potentially constraining the generalizability of these findings to the broader student population across Israel. Furthermore, there was an excess representation of women (reflecting the student population at the college). This representation may have contributed to the findings of higher levels of stress. The survey was conducted four months after 7 October, when participants resumed their regular routines and studies. It is reasonable to speculate that had the survey been administered shortly after 7 October, the stress levels among participants would have likely been significantly higher. Furthermore, this study does not encompass individual experiences of the war among participants. We lacked information about the war-related losses the students endured, which could be related to their stress levels. Nevertheless, this research contributes a significant analysis of stress within the distinctive context confronted by Israeli students.

## 5. Conclusions

This study sheds light on the significant impact of the ongoing war on the stress levels of Israeli college students, highlighting the need for targeted interventions and support services within higher education institutions. Acknowledging different stress responses based on gender, parental status, sleep quality, residency in conflict zones, and social media usage is crucial. Addressing these factors through tailored support programs, including psychological counseling, stress management workshops, and academic adjustments, can help mitigate the adverse effects of war-related stress on students’ well-being.

Furthermore, the findings underscore the importance of a comprehensive approach to trauma recovery, considering both individual and social factors. By enhancing the bond between students and their communities and providing support within familial, collegiate, and broader social networks, higher education institutions can play a pivotal role in fostering resilience and coping mechanisms among students during times of crisis. Future research should consider longitudinal designs among college students affected by war to capture changes in stress levels over time and explore additional factors influencing students’ resilience and coping strategies during wartime. Nonetheless, this study provides a foundational understanding of the stress dynamics within the unique context of the ongoing war, informing targeted interventions to support the mental well-being of Israeli college students.

## Figures and Tables

**Table 1:** Sample’s characteristics.

Characteristics	*n*	%
Gender:		
Male	175	28
Female	450	72
In a relationship	320	51
Have children	166	27
Jewish	501	80
Faculty:		
Social Sciences	357	57
Health Sciences	163	26
Computers and Management	105	17
Living in the conflict zone	265	42
Time spent on social media per day:		
Does not have/use any social media account	68	11
Up to one hour	101	16
Between 1 and 2 h	132	21
Approximately 2–3 h	158	25
More than 3 h	166	27
Sleep quality:		
Poor	50	8
Pretty poor	237	38
Pretty good	284	45
Very good	54	9

**Table 2:** The distribution of answers to the questionnaire focused on stress.

Statement	Seldom (%)	Sometimes (%)	Often (%)
I was worried because of something that happened unexpectedly	18	31	51
I felt that I was not able to control the important things in my life	35	33	33
I felt nervous and stressed	20	35	45
I felt [un]confident in my ability to handle my problems **	46	34	20
I felt that I was [not] succeeding (things were [not] working out for me) **	35	41	24
I felt like I could not cope with all the things I had to deal with	33	38	29
I could [not] control things that bothered me **	37	43	20
I felt that I was [not] in control of things **	38	40	22
I was angry due to matters that were out of my control	27	34	39
I felt many difficulties, and I could not overcome them	44	33	23

** Contrasting questions: The data are presented in reverse rank order.

**Table 3:** Relationships between study variables and stress.

Variable	Groups	Mean ± SD *	F/t/r	*p*
Gender	Male	2.61 ± 0.70	_t(623)_ = 7.89	<0.001
	Female	3.11 ± 0.72		
In a relationship	Yes	2.98 ± 0.80	t_(623)_ = 0.16	0.871
	No	2.97 ± 0.69		
Have children	Yes	3.05 ± 0.74	t_(623)_ = 4.50	<0.001
	No	2.75 ± 0.73		
Religion	Jewish	2.96 ± 0.78	t_(623)_ = 0.98	0.327
	Not Jewish	2.91 ± 0.57		
Faculty	Social Sciences	2.95 ± 0.76	F_(624)_ = 1.72	0.180
	Health Sciences	3.05 ± 0.69		
	Computers and Management	2.89 ± 0.78		
Age			r_p_ = −0.12	0.02
Sleep quality			r_s_ = −0.22	<0.001
Time spent on social media			r_p_ = 0.23	<0.001

* SD = standard deviation.

**Table 4:** Linear regression model results for predicting stress.

Variable	B	β	*p*
Gender _(0—male, 1—female)_	0.46	0.28	<0.001
Having children _(0—no, 1—yes)_	0.26	0.15	0.008
Age	0.00	0.03	0.554
Sleep quality	−0.22	−0.23	<0.001
Residency in a conflict zone _(0—no, 1—yes)_	0.13	0.09	0.024
Time spent on social media	0.08	0.12	0.004
Adjusted R square	0.19, *p* < 0.001		
F	22.79, *p* < 0.001		
N	557		

## Data Availability

The original contributions presented in the study are included in the article. Further inquiries can be directed to the corresponding author.
